# Efficacy of immune checkpoint inhibitors combined with bevacizumab in MSS/pMMR advanced colorectal cancer after first-line treatment failure

**DOI:** 10.3389/fonc.2024.1429095

**Published:** 2024-08-12

**Authors:** Xiaoqian Chen, Wenkui Li, Xiaogai Lei, Zhanhong Li, Qijing Guo, Xinfu Ma, Yushuang Luo, Liang Wang

**Affiliations:** ^1^ Department of Gastrointestinal Oncology Surgery, Affiliated Hospital of Qinghai University and Affiliated Cancer Hospital of Qinghai University, Xining, Qinghai, China; ^2^ Department of Emergency Medicine, The Second People's Hospital, Xining, Qinghai, China; ^3^ Gastroenterology Department, Qinghai Provincial Red Cross Hospital, Xining, Qinghai, China; ^4^ Gastroenterology Department, The Second People's Hospital, Xining, Qinghai, China; ^5^ Department of Oncology, Affiliated Hospital of Qinghai University and Affiliated Cancer Hospital of Qinghai University, Xining, Qinghai, China

**Keywords:** immune checkpoint inhibitors, bevacizumab, colorectal cancer, cytotoxic T lymphocytes, tumor-associated macrophages, cancer-associated fibroblasts

## Abstract

**Objective:**

To investigate the effects of a PD-1 inhibitor combined with a bevacizumab monoclonal antibody on tumor immune cells in patients with first-line treatment failure in MSS/pMMR advanced colorectal cancer.

**Methods:**

Control group consisted of 50 patients treated with the FOLFIRI combined with Bevacizumab regimen. The experimental group consisted of 60 patients treated with the Sintilimab combined with Bevacizumab regimen. By comparing the expression levels of CD8+ T lymphocytes, TAMs, and CAFs before and after treatment, short-term efficacy after treatment, and adverse drug reactions between the two groups, we comprehensively evaluated the impact of Sintilimab combined with Bevacizumab on patients with MSS/pMMR advanced colorectal cancer who failed first-line treatment.

**Results:**

There was a statistically significant difference in the percentage of CD8+ T lymphocytes, TAMs, and CAFs before and after treatment between the two groups (*P*<0.05);Immunohistochemical scoring of CD8+ T lymphocytes, TAMs, and CAFs showed significant differences between the groups post-treatment *(P<0.05*). The experimental group demonstrated statistically significant differences in immunohistochemical scoring of CD8+ T lymphocytes, TAMs, and CAFs before and after treatment (*P<0.05*). There was a statistically significant difference in the therapeutic effect between the two groups of tumors (*P*<0.05). The experimental group had greater PFS, mPFS, ORR, and DCR than did the control group. There was no statistically significant difference in the occurrence rate of drug-related adverse reactions after treatment between the two groups (*P*>0.05). The results of the Cox proportional hazards model analysis indicate that age, gender, and group are independent risk factors affecting MSS/pMMR advanced colorectal cancer patients treated with second-line therapy in this study. Patients aged ≤60 years, male patients, and those in the experimental group showed better treatment responses in this study.

**Conclusion:**

By administering immune checkpoint inhibitors in combination with bevacizumab to patients with advanced colorectal cancer with MSS/pMMR disease for whom first-line treatment failed, not only did the patients’ prognosis improve, but the adverse drug reactions were also safe and controllable.

## Introduction

Colorectal cancer is a common malignant tumor of the digestive tract in China and has a high incidence and mortality rate. According to the 2020 GLOBOCAN statistics, there are more than 1.9 million newly diagnosed cases of colorectal cancer worldwide, with approximately 935,000 deaths from colorectal cancer. It ranks third in the incidence spectrum of malignant tumors and second in the mortality spectrum, accounting for approximately one-tenth of the incidence and mortality rates of cancer (1, 2). Studies (3, 4) have shown that the efficacy of second-line treatment after first-line treatment for advanced colorectal cancer is low. The identification of treatment options for advanced colon cancer patients for whom first-line treatment has failed remains a hot topic in current clinical research.

Currently, immune checkpoint inhibitors (ICIs) have become the first-line standard treatment for metastatic colorectal cancer (mCRC) patients with high microsatellite instability/mismatch repair deficiency (MSI-H/dMMR). However, for microsatellite stable (MSS) colon cancer, the efficacy of ICIs treatment is minimal, mainly because this type of tumor is a ‘cold tumor’ with almost no lymphocyte infiltration. However, antiangiogenic drugs can improve the immune microenvironment by promoting the entry of more immune cells into the immune microenvironment, thereby exerting antitumor effects. The REGONIVO and REGOTORI studies (5, 6) have confirmed that the combination of ICIs and antiangiogenic drugs benefits MSS advanced colon cancer patients who have failed repeated treatments after third-line therapy. The effects of combinations of different ICIs and antiangiogenic agents may not be completely the same. The efficacy and safety of the combination of the above two drugs in MSS mCRC patients who have failed first-line treatment have not been reported.

Cytotoxic T lymphocytes (CD8+ T cells) are the main effector cells involved in tumor immunity. Studies (7) have shown that patients with high-density tumor antigen-specific CD8+ T lymphocytes at the invasive tumor edge are more likely to benefit from treatment with programmed death 1 (PD-1)/programmed cell death-ligand 1 (PD-L1) inhibitors. Tumor-associated macrophages (TAMs) and cancer-associated fibroblasts (CAFs) are the two main stromal cell types in the tumor microenvironment (TME). Studies (8, 9) have shown that an increase in the number of TAMs and CAFs, as well as their interaction, leads to changes in the tumor immune microenvironment. This not only enhances their protumor effects but also has a close relationship with tumor metastasis and recurrence, affecting the efficacy of ICIs treatment. There is limited research on the effects of the combination of PD-1/PD-L1 inhibitors and anti-vascular endothelial growth factor (VEGF)/vascular endothelial growth factor receptor (VEGFR) drugs on tumor immune cells.

Although the traditional chemotherapy regimen has certain efficacy in the treatment of advanced colorectal cancer, it has more adverse reactions and affects the survival quality of patients. Moreover, while killing tumor cells, traditional chemotherapeutic regimens will also bring different degrees of damage to immune cells, which will then lead to the reduced ability of the body’s immune system to recognize and kill tumor cells, increase the recurrence and metastasis after colorectal cancer treatment, and is not conducive to the prognosis of patients (10). About 95% of colorectal cancers are MSS/proficient mismatch repair (pMMR), which have lower levels of tumor lymphocyte infiltration and tumor mutational burden (TMB), and are therefore insensitive to the treatment of immune checkpoint inhibitors (11). In the KEYNOTE-028 and KEYNOTE-016 studies (12, 13), no significant treatment response was observed when Pembrolizumab monotherapy was applied to treat advanced MSS/pMMR-type metastatic colorectal cancer. The method of enhancing the efficacy of immune checkpoint inhibitors in MSS/pMMR-type mCRC patients is currently a critical area of exploration. Combination therapies may potentially transform some immune-resistant “cold tumors” into immune-sensitive “hot tumors.” The METIMMOX study (14) showed that using Oxaliplatin combined with Nivolumab as first-line treatment for MSS/pMMR-type metastatic colorectal cancer resulted in a median progression-free survival (mPFS) of 6.6 months and an objective response rate (ORR) of 32% within a median follow-up period of 6.4 months. In the chemotherapy control group, mPFS was 5.6 months, and ORR was 23%. The AtezoTRIBE study (15) showed that FOLFOXIRI combined with Atezolizumab as first-line treatment for MSS/pMMR-type metastatic colorectal cancer resulted in an mPFS of 12.9 months within a median follow-up period of 19.9 months, compared to an mPFS of 11.4 months in the control group. A study from China (16) indicated that Fruquintinib combined with Sintilimab was effective in treating advanced MSS/pMMR-type metastatic colorectal cancer, with an mPFS of 6.7 months, an ORR of 18.18%, and a disease control rate (DCR) of 63.64%. In the Fruquintinib monotherapy group, mPFS was 4.4 months, ORR was 9.09%, and DCR was 45.45%.In this study, we prospectively enrolled 110 patients with MSS/pMMR advanced colon cancer who failed first-line treatment, with 60 patients in the experimental group receiving bevacizumab combined with sintilimab treatment and 50 patients in the control group receiving FOLFIRI combined with bevacizumab treatment. We observed drug-related adverse reactions and graded according to CTCAE (17) in both groups, simultaneously analyzing changes in tumor CD8+ T cells, TAMs, CAFs counts, immunohistochemistry score changes before and after treatment, and their correlation with treatment efficacy. We evaluated short-term prognoses and identified independent risk factors for both groups, clarifying that the combination of PD-1 inhibitors and VEGF inhibitors can improve the tumor immune tolerance microenvironment of MSS/pMMR advanced colon cancer, enhance the effectiveness of immune therapy, provide more clinical evidence for subsequent large-sample clinical studies, and establish a theoretical basis for personalized immunotherapy of MSS/pMMR colon cancer.

## Materials and methods

### General information

A total of 110 patients with MSS/pMMR advanced colorectal cancer who failed first-line treatment were selected using a random number table method from October 2021 to June 2023 at the Affiliated Hospital of Qinghai University. There were 60 patients in the experimental group, including 36 males and 24 females; aged between 50 and 75 years, with an average age of 62.23 ± 7.49 years; 8 patients in stage III, 52 patients in stage IV; 30 patients with poorly differentiated tumors, 22 patients with moderately differentiated tumors, 8 patients with well-differentiated tumors, with an average BMI of 20.58 ± 2.03 kg/m^2^; and 36 patients with colon cancer, 24 patients with rectal cancer. There were 50 patients in the control group, including 30 males and 20 females; aged between 50 and 75 years, with an average age of 61.20 ± 7.74 years; 4 patients in stage III, 46 patients in stage IV; 35 had poorly differentiated tumors; 10 had moderately differentiated tumors; 5 had well-differentiated tumors; the average BMI was 20.17 ± 1.65 kg/m^2^; 25 patients had colon cancer; and 25 patients had rectal cancer. There was no significant difference in the baseline data between the two groups (*P*>0.05), as shown in **Table 1**. This study was approved by the hospital’s ethics committee, and informed consent was obtained from the patients.

**Table 1:** Comparison of baseline data between the two groups (%).

	Experimental Group (n=60)	Control Group (n=50)	*P-*value
Gender (Cases)			
Male	36 (60.00)	30 (60.00)	1.000
Female	24 (40.00)	20 (40.00)	
Age (Years)	62.23 ± 7.49	61.20 ± 7.74	0.480
Clinical Stage			
Stage III	8 (13.30)	4 (8.00)	0.366
Stage IV	52 (86.70)	46 (92.00)	
Stage IV			
Hepatic metastases	48	43	0.532
Bone metastases	3	1	
Pulmonary metastasis	1	1	
Brain metastases	0	1	
Degree of Differentiation			
Low Differentiation	30 (50.00)	35 (70.00)	0.091
Moderate Differentiation	22 (36.70)	10 (20.00)	
High Differentiation	8 (13.30)	5 (10.00)	
BMI (Kg/m^2^)	20.58 ± 2.03	20.17 ± 1.65	0.256
Colon Cancer Rectal Cancer	36 (60.00) 24 (40.00)	25 (50.00) 25 (50.00)	0.293

### Inclusion, exclusion, and withdrawal criteria

#### Inclusion criteria

① Patients who were pathologically confirmed to have colorectal cancer, including signet ring cell carcinoma and mucinous adenocarcinoma, met the inclusion criteria, and hepatoid adenocarcinoma, undifferentiated carcinoma, and no other tumors were excluded.

② The disease is unresectable, locally advanced or metastatic; the RAS or BRAF gene is mutated; patients who have received first-line oxaliplatin combined with bevacizumab treatment and failed first-line treatment; and patients who have not previously used PD-L1 inhibitors or PD-1 inhibitors.

③ Patients aged older than 18 years and younger than 80 years.

④ Imaging evaluation: According to the RECIST criteria, there is at least one measurable lesion.

⑤ Eastern Cooperative Oncology Group, ECOG (18) performance status of 0 or 1.

⑥Expected survival ≥12 weeks.

⑦ All the patients had sufficient organ and bone marrow functions.

⑧ Women of childbearing potential or male partners of women of childbearing potential were required to use effective contraception throughout and for 6 months following the treatment period.

⑨Patients who provided written informed consent and who were able to abide by the relevant procedures stipulated in the study.

#### Exclusion criteria

① Previous exposure to any antibody or drug therapy against PD-1, PD-L1, PD-L2, CD137, CTLA-4, or any other antibody or drug with T-cell costimulation or a checkpoint pathway as the specific target; bone metastasis at risk of paraplegia; and wild-type RAS or BRAF gene.

② Signs of active bleeding in known lesions (including hematemesis and melena in the first 2 weeks at random); In the first 3 months, there was a gastrointestinal bleeding event of grade 3 or above (NCICTCAEv5.0) that required blood transfusion, invasive intervention or hospitalization; severe hemorrhagic disease; or other conditions that led to a high risk of bleeding.

③ Significant malnutrition.

④ Receipt or planned receipt of a live attenuated vaccine within 4 weeks prior to randomization or during the study period. Known acute or chronic active hepatitis B virus or acute or chronic active hepatitis C virus infection, active tuberculosis, syphilis infection requiring treatment, history of human immunodeficiency virus (HIV) infection (i.e., positive HIV antibody), or severe infections that are active or poorly controlled clinically. The history of primary immunodeficiency is known.

⑤ Symptomatic congestive heart failure (New York Heart Association Class II-IV), symptomatic or poorly controlled arrhythmia, or left ventricular ejection fraction (LVEF) < 50% as indicated by echocardiography. Any arterial thromboembolic event within 6 months prior to randomization, including myocardial infarction, unstable angina, cerebrovascular accident, transient ischemic attack, renal artery embolism, gastrointestinal artery embolism, etc. Any history of deep vein thrombosis, pulmonary embolism, or other severe thromboembolism within 3 months prior to randomization (thrombosis related to implantable venous infusion port or catheter, or superficial vein thrombosis is not considered “severe” thromboembolism).

⑥ Current anticoagulant treatment with low molecular weight heparin, warfarin, or similar medications.

⑦ Known or suspected autoimmune diseases (such as systemic lupus erythematosus, myasthenia gravis, vasculitis, etc.) or a history of these diseases within the past 2 years (patients with vitiligo, psoriasis, alopecia, or Graves’ disease not requiring systemic treatment within the past 2 years, patients with hypothyroidism or autoimmune thyroiditis requiring only thyroid hormone replacement therapy, and patients with type 1 diabetes requiring only insulin replacement therapy may be enrolled).

⑧ Known history of allogeneic organ transplantation or allogeneic hematopoietic stem cell transplantation.

⑨ Pregnant (positive urine or serum pregnancy test) or breastfeeding female patients.

⑩severe primary diseases of the liver, kidney, hematopoietic system, abnormal liver function (aspartate aminotransferase or alanine aminotransferase greater than 1.5/2 times the upper limit of normal), and elevated creatinine levels exceeding the upper limit of normal.

#### Criteria for withdrawal

① Severe toxicity or intolerance to treatment but should be analyzed and recorded as adverse reactions;

② Subjects who requested to withdraw from the trial themselves or whose investigator believed that it was medically necessary to withdraw from the study.

### Methods

The control group was treated with the FOLFIRI combined with Bevacizumab regimen. On the first day of each cycle, the following drugs were administered via intravenous infusion: Bevacizumab (National Drug Approval Number: S20200013, Manufacturer: Xinda Biologics (Suzhou) Co., Ltd., Specification: 4ml:100mg), at a dose of 5 mg/kg, fully mixed in 250 mL of 0.9% sodium chloride injection (National Drug Approval Number: H37022918, Manufacturer: Shandong Kelun Pharmaceutical Co., Ltd., Specification: 250 mL:2.25 g/bottle) and infused intravenously over 60 minutes. Irinotecan (National Drug Approval Number: H20084572, Manufacturer: Qilu Pharmaceutical Co., Ltd., Specification: 5ml:0.1g), at a dose of 180 mg/m², fully mixed in 250 mL of 5% glucose injection (National Drug Approval Number: H20023879, Manufacturer: Shandong Kelun Pharmaceutical Co., Ltd., Specification: 250 mL:12.5 g/bottle) and infused intravenously over 30-90 minutes. Calcium folinate (National Drug Approval Number: H20020609, Manufacturer: Jiangsu Hengrui Medicine Co., Ltd., Specification: 3ml:30mg), at a dose of 400 mg/m², fully mixed in 250 mL of 5% glucose injection (National Drug Approval Number: H20023879, Manufacturer: Shandong Kelun Pharmaceutical Co., Ltd., Specification: 250 mL:12.5 g/bottle) and infused intravenously over 30-90 minutes. Fluorouracil (National Drug Approval Number: H20223398, Manufacturer: Sichuan Huiyu Pharmaceutical Co., Ltd., Specification: 10ml:0.25g), at a dose of 400 mg/m², fully mixed in 250 mL of 5% glucose injection (National Drug Approval Number: H20023879, Manufacturer: Shandong Kelun Pharmaceutical Co., Ltd., Specification: 250 mL:12.5 g/bottle) and infused intravenously over 30-90 minutes. Fluorouracil (National Drug Approval Number: H20223398, Manufacturer: Sichuan Huiyu Pharmaceutical Co., Ltd., Specification: 10ml:0.25g), at a dose of 1200 mg/m², fully mixed in 200 mL of 5% glucose injection (National Drug Approval Number: H20023879, Manufacturer: Shandong Kelun Pharmaceutical Co., Ltd., Specification: 250 mL:12.5 g/bottle) and continuously infused via chemotherapy micro-infusion pump over 48 hours. After a 21-day break, the next treatment cycle began. A total of 4 cycles were administered (19).

The experimental group was treated with the Sintilimab combined with Bevacizumab regimen. The following drugs were administered via intravenous infusion: Sintilimab (National Drug Approval Number: S20180016, Manufacturer: Xinda Biologics (Suzhou) Co., Ltd., Specification: 10ml:100mg), at a daily dose of 200mg, fully mixed in 100 mL of 0.9% sodium chloride injection (National Drug Approval Number: H20013026, Manufacturer: Shandong Kelun Pharmaceutical Co., Ltd., Specification: 100 mL:0.9 g/bottle) and infused intravenously over 30-60 minutes. Bevacizumab (National Drug Approval Number: S20200013, Manufacturer: Xinda Biologics (Suzhou) Co., Ltd., Specification: 4ml:100mg), at a daily dose of 7.5 mg/kg, fully mixed in 250 mL of 0.9% sodium chloride injection (National Drug Approval Number: H37022918, Manufacturer: Shandong Kelun Pharmaceutical Co., Ltd., Specification: 250 mL:2.25 g/bottle) and infused intravenously over 60 minutes. Each cycle lasted 21 days, and a total of 4 cycles were administered (20).

Both groups received prophylactic anti-allergy and antiemetic treatment 30 minutes before the start of chemotherapy: Dexamethasone sodium phosphate injection (National Drug Approval Number: H41021924, Manufacturer: Shanghai Baiyang Pharmaceutical Co., Ltd., Specification: 1ml:5mg) via intramuscular injection. Palonosetron injection (National Drug Approval Number: H20194015, Manufacturer: Kunming Jida Pharmaceutical Co., Ltd., Specification: 1.5ml:0.075mg) via intravenous injection.

During treatment, patients were closely monitored for symptoms, signs, and vital indicators. Any adverse toxic side effects were meticulously recorded and graded according to the Common Terminology Criteria for Adverse Events (CTCAE) v5.0, ranging from Grade 1 to 5. Seven days after the end of treatment, routine blood tests and liver, kidney, and heart function tests were repeated. Patients were followed up regularly after discharge.

#### Observation indicators

(1) Primary Endpoint Indicators: Progression-Free Survival (PFS): Defined as the time from randomization to tumor progression or death from any cause (whichever occurs first). mPFS refers to the time at which 50% of patients have achieved progression-free survival.
(2) Secondary Endpoint Indicators: Treatment Efficacy Evaluation: Based on the Response Evaluation Criteria in Solid Tumors (RECIST) (21), the efficacy of the treatment in both groups was assessed, including the following indicators: Complete Remission (CR): Disappearance of all tumor target lesions with no new lesions, maintained for at least 4 weeks. Partial Remission (PR): A reduction in the sum of the longest diameter of baseline target lesions by more than 30% with no change within 4 weeks. Stable Disease (SD): Reduction less than 30% and growth less than 20% with no change within 4 weeks. Progression Disease (PD): An increase in the sum of the longest diameter of target lesions by more than 20% or the appearance of new lesions. ORR: The percentage of patients achieving CR and PR. DCR: The percentage of patients achieving CR, PR, and SD.
(3) Exploratory Endpoint Indicators: Analysis of the impact of immune checkpoint inhibitors combined with anti-angiogenesis drugs on the expression of CD8+ T cells, TAMs, and CAFs in the tumor microenvironment.
(4) Safety Evaluation: Drug-related adverse reaction were assessed according to the NCI Common Terminology Criteria for Adverse Events (NCI-CTCAE) version 5.0.

In this study, all treated patients had sufficient tumor tissue samples collected by the same endoscopy center team using electronic colonoscopy before treatment and after 4 cycles of combination therapy. These samples were immediately sent to the same pathology testing center team for analysis.

#### Detection of CD8+ T cells, tumor-associated macrophages, and cancer-associated fibroblasts expression

Immunohistochemistry Specific Steps:

- Deparaffinization with xylene and rehydration through a graded alcohol series.
- Soaking in 3% hydrogen peroxide-methanol solution at room temperature for 10 minutes to remove endogenous peroxidase.
- High-pressure antigen retrieval: Place 1500ml of pH 9.0 Tris/EDTA buffer in a pressure cooker, bring to a boil, place the slices on a stainless-steel slide rack in the cooker, time for 2 minutes after the steam releases, then remove from heat and cool naturally.
- Blocking with normal goat serum working solution at room temperature for 2 hours to block non-specific antigen sites.
- Adding the primary antibody and incubating overnight at 4°C.
- Adding horseradish peroxidase-labeled goat anti-mouse/rabbit IgH polymer and incubating at 37°C for 15 minutes.
- Adding DAB chromogen, stopping the reaction when brown-yellow color appears under the microscope.
- Counterstaining with hematoxylin, differentiating with hydrochloric acid alcohol, bluing, and mounting with neutral resin.

### Detection of the expression of CD8+ T cells, TAMs, and CAFs through immunohistochemistry

#### Interpretation of immunohistochemistry results

a. CD8-positive cells were located on the lymphocyte membrane, and the staining intensity was scored as follows: no staining, 0 points; light yellow, 1 point; brown‒yellow, 2 points; and brown, 3 points. The staining area was assessed by counting the lymphocytes in the field of view; <5% of the lymphocytes were stained at 0 points, 5%-25% of the lymphocytes were stained at 1 point, 25%-50% of the lymphocytes were stained at 2 points, and >50% of the lymphocytes were stained at 3 points. The two scores were multiplied: <2 points are negative, and ≥2 points are positive.

b. CAF expression localization: The specific protein α-smooth muscle actin (α-SMA) of CAFs was used as the immunoenzyme target antigen, with α-SMA-positive cells in the tumor stroma representing CAFs. Ten high-power fields (×400) with approximately 200 cells were randomly selected, and the percentage of positive cells among the observed cells was scored as follows: 1 point (≤10% positive cells), 2 points (10%< positive cells ≤50%), 3 points (50%< positive cells ≤75%), and 4 points (75%< positive cells). The staining intensity was scored as follows: 0 points (no staining), 1 point (light yellow), 2 points (brown‒yellow), and 3 points (brown). The two scores were multiplied: 0-3 points (-), 4-5 points (+), 6-7 points (++), and ≥8 points (+++), where +-+++ indicates positive expression.

c. TAM expression localization: CD68, CD163, and HLA-DR antibodies were used to label human macrophages, M2-like TAMs, and M1-like TAMs, respectively, to further study macrophage polarization. The area with the greatest immune response was selected for quantification, and the staining results were scored based on the percentage of positive cells and the intensity of staining, known as the immunoreactive score (IRS). The scores for the percentage of positive cells were as follows: 0 points (unstained cells); 1 point (<25% of cells stained); 2 points (25%~75% of cells stained); and 3 points (>75% of cells stained). The staining intensity scores were as follows: 0 points (negative); 1 point (weakly positive); 2 points (moderately positive); and 3 points (strongly positive). IRS = score for the percentage of positive cells × score for staining intensity. IRS=0~2 was defined as negative/low expression; IRS=3~9 was defined as positive/high expression.

The study data were analyzed using SPSS 25.0 software. Data following a normal distribution were expressed as mean and standard deviation $(\overset{¯}{X}\pm S)$ , and comparisons were made using the t-test or t′-test for two independent samples. For paired samples, paired samples t-test was applied to data. Data not following a normal distribution were represented as *M (Q_L_−Q_U_)* and compared using the rank-sum test. Qualitative data were analyzed using the chi-square test or the rank-sum test. The Cox proportional hazards model was used to analyze the impact of multiple risk factors on survival outcomes and to explore the relative importance of these factors. When *P < 0.05*, the difference was considered to be statistically significant. GraphPad Prism 9.00 software was used for graphic statistics.

## Results

### Comparison of the percentages of CD8+ T lymphocytes, TAMs, and CAFs before and after treatment in the two groups of patients

**Figure 1:**
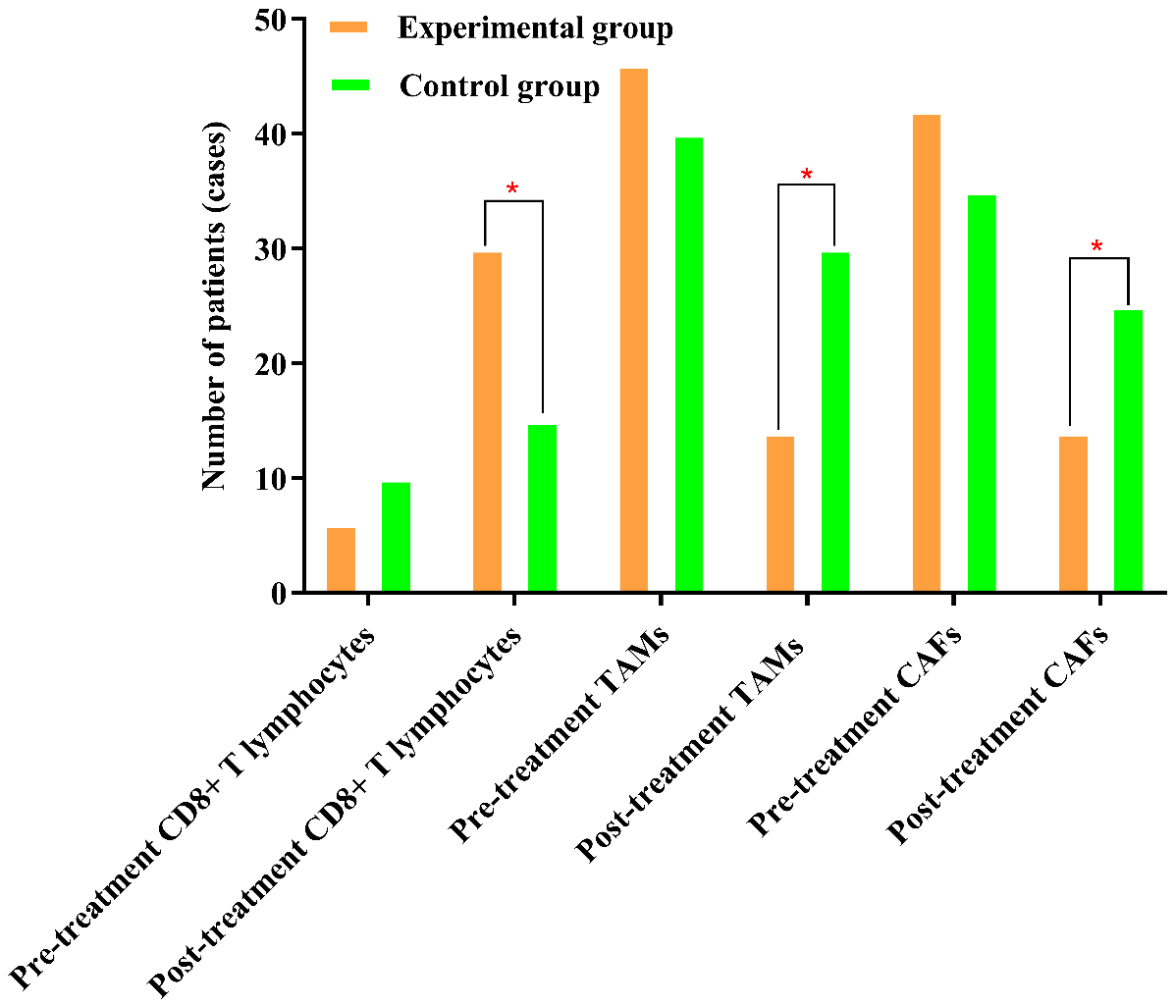
The percentages of TAMs and CAFs in the experimental group were significantly lower after treatment than before treatment. The symbol “*” denotes statistical significance between the two groups (*P<0.05*).

The percentage of CD8+ T lymphocytes in the experimental group was significantly greater after treatment than before treatment (*P*<0.05). The percentages of TAMs and CAFs in the experimental group were significantly lower after treatment than before treatment (*P*<0.05). The results are shown in **Table 2** and **Figure 1**.

**Table 2A:** Comparisons of the percentages of CD8+ T lymphocytes, TAMs, and CAFs before and after treatment in the two groups of patients [cases (%)].

Group	Number of Cases	CD8+ T Lymphocytes		TAMs		CAFs	
		Before Treatment	After treatment	Before Treatment	After treatment	Before Treatment	After treatment
Experimental group	60	6(10.00)	30(50.00)	46(76.70)	14(23.30)	42(70.00)	14(23.30)
Control group	50	10(20.00)	15(30.00)	40(80.00)	30(60.00)	35(70.00)	25(50.00)
*P-*value		0.139	0.033	0.673	0.001	1.000	0.003

**TABLE 2B:** Comparison of immunohistochemistry scores before and after treatment in the two groups of patients [scores].

	Number of Cases	CD8+ T Lymphocytes	TAMs	CAFs
Before Treatment				
Experimental group	60	0.51 ± 0.77	3.46 ± 1.01	4.23 ± 1.26
Control group	50	0.54 ± 0.81	3.06 ± 0.76	3.34 ± 1.17
*P-*value		0.878	0.052	0.052
After Treatment				
Experimental group	60	1.73 ± 0.86	2.03 ± 0.75	2.55 ± 1.06
Control group	50	0.64 ± 0.92	2.84 ± 1.47	3.06 ± 1.07
*P-*value		<0.001	0.001	0.014

**TABLE 2C:** Comparison of immunohistochemistry scores before and after treatment within the two groups of patients [scores].

Group	Number of Cases	CD8+ T Lymphocytes		TAMs		CAFs	
		Before Treatment	After treatment	Before Treatment	After treatment	Before Treatment	After treatment
Experimental group	60	0.51 ± 0.77	1.73 ± 0.86	3.46 ± 1.01	2.03 ± 0.75	4.23 ± 1.26	2.55 ± 1.06
*P-*value		<0.001		<0.001		<0.001	
Control group	50	0.54 ± 0.81	0.64 ± 0.92	3.06 ± 0.76	2.84 ± 1.47	3.34 ± 1.17	3.06 ± 1.07
*P-*value		0.096		0.231		0.056	

### Comparison of immunohistochemistry scores before and after treatment in the two groups of patients

**Figure 2:**
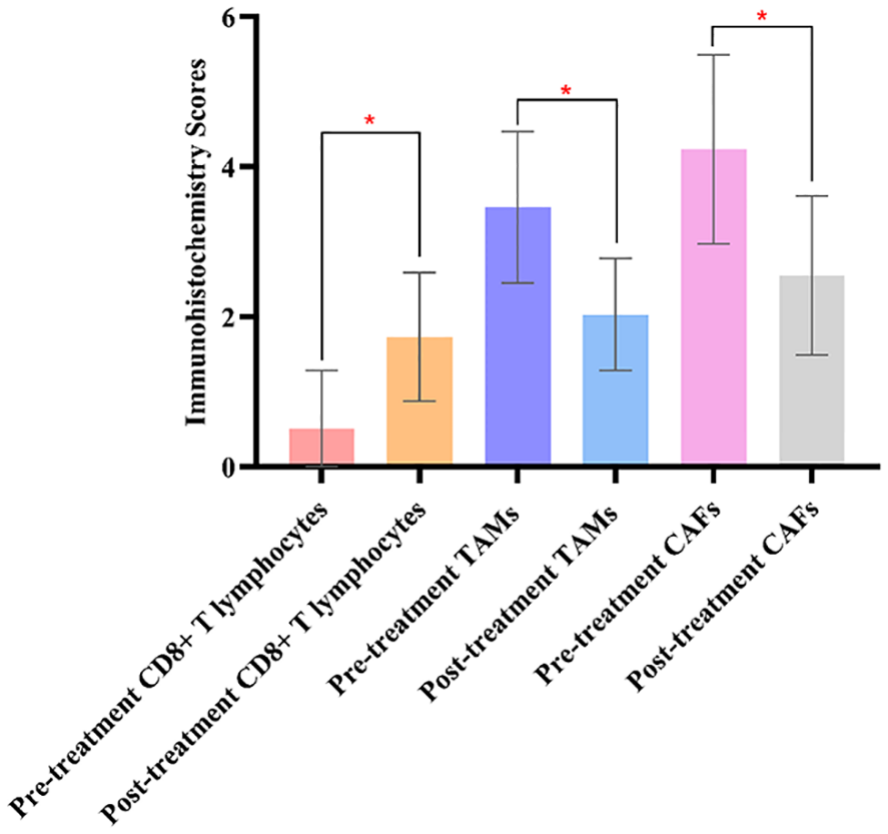
Comparing the immunohistochemistry scores of CD8+ T lymphocytes, TAMs, and CAFs in the experimental groups before and after treatment, there were significant differences in the experimental group. The symbol “*” denotes statistical significance between the two groups (*P<0.05*).

Before treatment, the comparison of immunohistochemistry scores for CD8+ T lymphocytes, TAMs, and CAFs between the two groups showed no statistically significant differences (*P>0.05*), indicating the comparability of the two groups in terms of immunohistochemistry scores. After treatment, there were significant differences in the immunohistochemistry scores for CD8+ T lymphocytes, TAMs, and CAFs between the two groups (*P<0.05*). The results are shown in **Figure 2**.

### Comparison of immunohistochemistry scores before and after treatment within the two groups of patients

The comparison of immunohistochemistry scores for CD8+ T lymphocytes, TAMs, and CAFs before and after treatment showed statistically significant differences in the experimental group (*P<0.05*). However, there were no significant differences in the control group before and after treatment (*P>0.05*).

### Comparison of tumor treatment effects between the two groups

**Figure 3:**
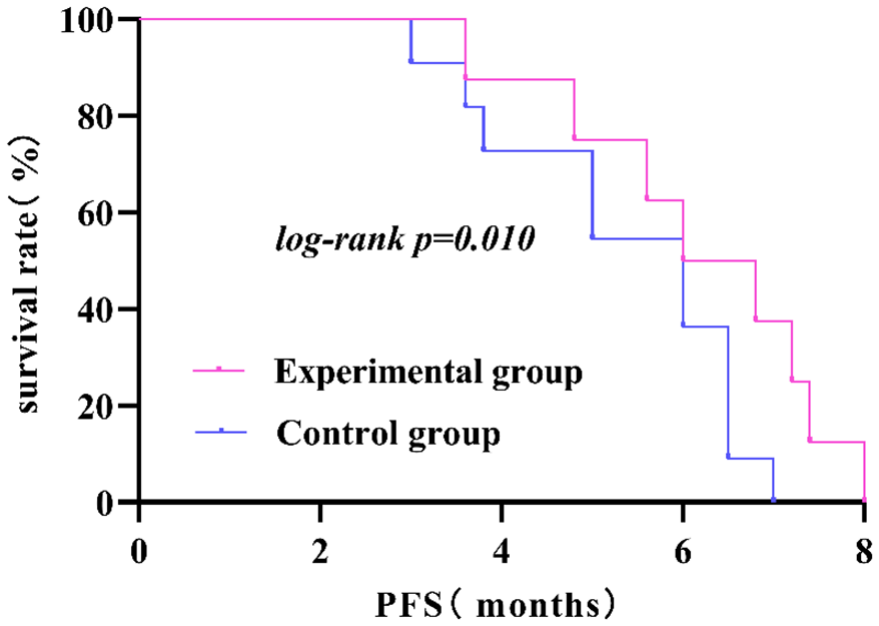
The PFS in the experimental group was significantly longer than that in the control group (*P<0.05*).

There were no cases of CR in either group; after treatment evaluation, the experimental group had 16 cases of PR, with an ORR of 26.70%; the control group had 5 cases of PR, with an ORR of 10.00%, and this difference was statistically significant (*P*<0.05). Although both the experimental and control groups had some patients with SD and PD, this difference was not statistically significant (*P*>0.05); the ORR and DCR in the experimental group were greater than those in the control group, but only the difference in the DCR between the two groups was not statistically significant (*P>0.05*). In terms of PFS, the PFS of the experimental group was 5.04 ± 1.83 months, and that of the control group was 4.69 ± 1.30 months; however, these differences were not statistically significant (*P*>0.05). The mPFS was 5 months in the experimental group and 4 months in the control group. The results are shown in **Table 3** and **Figure 3**.

**Table 3:** A comparison of tumor treatment effects between the two groups.

Group	Number of cases	CR (cases)	PR (cases)	SD (cases)	PD (cases)	ORR[cases (%)]	DCR[cases (%)]	PFS (Months)	mPFS (Months)
Experimental group	60	0	16	12	32	16(26.70)	28(46.70)	5.04 ± 1.83	5
Control group	50	0	5	10	35	5(10.00)	15(30.00)	4.69 ± 1.30	4
*P*-value		–	0.023	1.000	0.073	0.023	0.073	0.247	–

### Comparison of drug-related adverse reactions between the two groups post-treatment according to CTCAE

**Figure 4:**
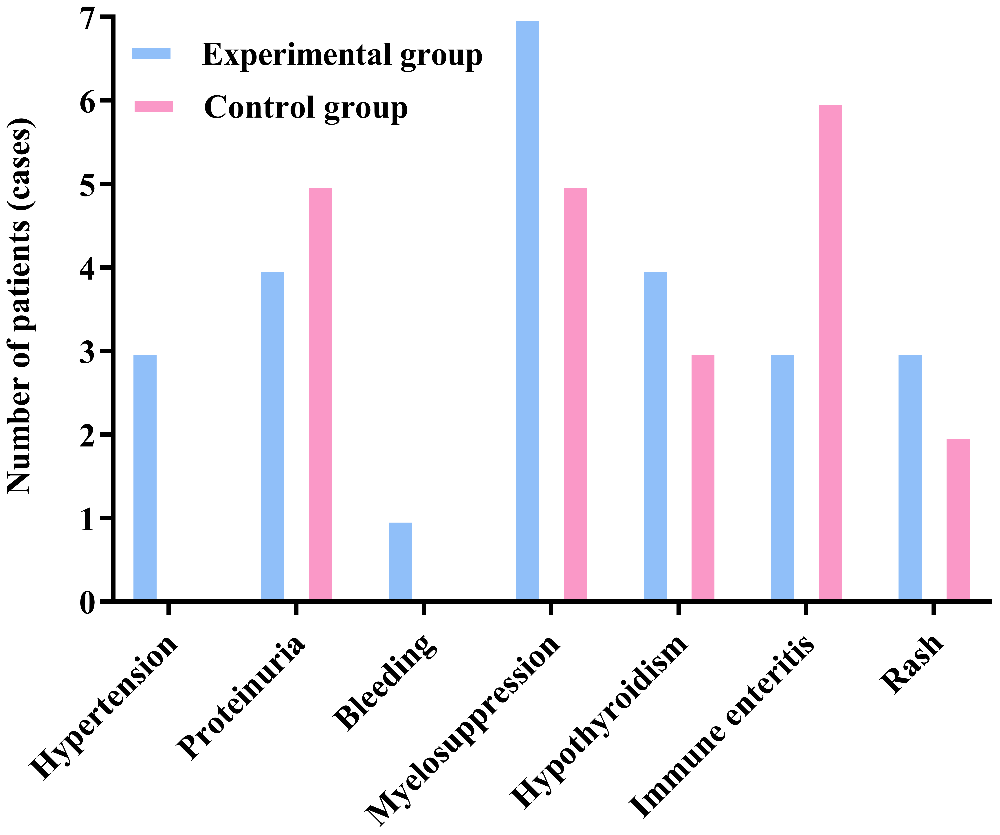
There was no significant difference in the incidence of adverse reactions between the two groups (*P>0.05*).

After treatment, there was no statistically significant difference in the incidence of drug-related adverse reactions such as hypertension, proteinuria, gastrointestinal perforation, bleeding, arterial thrombosis, Myelosuppression, immune pneumonitis, immune hepatitis, hypothyroidism, immune enteritis, and rash between the two groups (*P>0.05*). The results are shown in **Table 4** and **Figure 4**.

**Table 4A:** Comparison of the incidence of drug-related adverse reactions in patients in the two groups [cases (%)].

Group	Number of cases	Hypertension	Proteinuria	Gastrointestinal perforation	Bleeding	Arterial thrombosis	Myelosuppression
Experimental group	25	3 (5.00)	4 (6.70)	0 (0.00)	1 (1.70)	0 (0.00)	7 (11.70)
I		2 (3.30)	2 (3.35)	0 (0.00)	1 (1.70)	0 (0.00)	3 (5.00)
II		1 (1.70)	2 (3.35)	0 (0.00)	0 (0.00)	0 (0.00)	4 (6.70)
Control group	21	0 (0.00)	5 (10.00)	0 (0.00)	0 (0.00)	0 (0.00)	5 (10.00)
I		0 (0.00)	3 (6.00)	0 (0.00)	0 (0.00)	0 (0.00)	3 (6.00)
II		0 (0.00)	2 (4.00)	0 (0.00)	0 (0.00)	0 (0.00)	2 (4.00)
*P*-value	0.972	0.249	0.729	-	1.000	-	1.000

**TABLE 4B:** Comparison of the incidence of drug-related adverse reactions in patients in the two groups [cases (%)].

Group	Number of cases	Immune pneumonia, Immune Hepatitis	Hypothyroidism	Immune enteritis	Rash
Experimental group	25	0 (0.00)	4 (6.70)	3 (5.00)	3 (5.00)
I		0 (0.00)	2 (3.35)	2 (3.30)	3 (5.00)
II		0 (0.00)	2 (3.35)	1 (1.70)	0 (0.00)
Control group	21	0 (0.00)	3 (6.00)	6 (12.00)	2 (4.00)
I		0 (0.00)	2 (4.00)	5 (10.00)	2 (4.00)
II		0 (0.00)	1 (2.00)	1 (2.00)	0 (0.00)
*P*-value	0.972	-	1.000	0.295	1.000

### COX regression model analysis of prognostic factors influencing second-line treatment in MSS/pMMR advanced colorectal cancer patients

**Figure 5:**
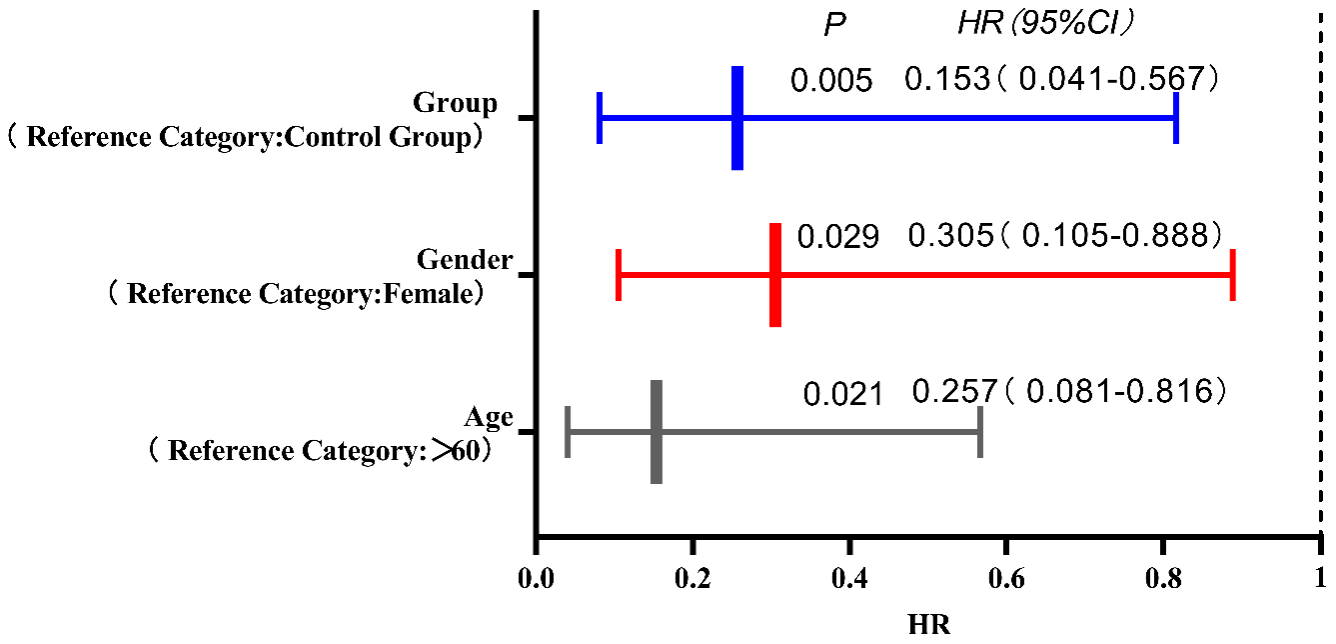
The COX regression model analysis showed that age, gender, and grouping were independent risk factors affecting the prognosis of MSS/pMMR advanced colorectal cancer patients undergoing second-line treatment (*P<0.05*).

The COX regression model analysis showed that age, gender, and grouping were independent risk factors affecting the prognosis of MSS/pMMR advanced colorectal cancer patients undergoing second-line treatment (*P<*0.05). The results are shown in **Table 5** and **Figure 5**.

**Table 5:** COX regression model analysis of prognostic factors influencing second-line treatment in MSS/pMMR advanced colorectal cancer patients.

Variable	*β*	*S.E.*	*Wald χ^2^*	*P*	*HR*	*95% CI*
Age	-1.878	0.669	7.880	0.005	0.153	0.041-0.567
Gender	-1.188	0.545	4.744	0.029	0.305	0.105-0.888
Group	-1.360	0.590	5.311	0.021	0.257	0.081-0.816
Degree of Differentiation						
Moderate Differentiation	-0.177	0.525	0.050	0.823	0.889	0.318-2.487
High Differentiation	-0.293	0.830	0.125	0.724	0.746	0.147-3.795
Clinical Stage						
Stage IV	-3.574	2.363	2.287	0.130	0.028	0.000-2.879

Using PFS as the dependent variable, the other variables were defined as follows: survival status (event-defined death = 1; 2 = survival status); independent variables: age (≤60 = 1, reference: >60 = 2), gender (male = 1, reference: female = 2), grouping (Experimental group = 1, reference: control group = 2), differentiation degree (poor differentiation = 1, moderate differentiation = 2, reference: dummy variable setting - high differentiation = 3), clinical stage (reference: dummy variable setting - stage III = 3, stage IV = 4).

## Discussion

Currently, more than 50% of colorectal cancer patients are diagnosed at an advanced stage or with distant metastasis. In clinical practice, simple chemotherapy or combination with molecular targeted therapy is often used as the main treatment for these patients. Although studies (3, 4, 22–24) have shown better clinical benefits for patients, there are still considerable limitations in terms of prolonging survival. For example, the tolerance of chemotherapy in advanced CRC patients who have previously received second-line treatment has decreased to varying degrees, especially in elderly and frail patients, with more obvious of chemotherapy drug toxicity and complications. Therefore, it is still very important in clinical practice to continue exploring safer and more effective treatment options for advanced CRC patients who have failed first-line treatment (25).

Breakthrough progress has been made in the first-line treatment of MSI-H/dMMR mCRC through the application of ICIs. The KEYNOTE-177 study (26) evaluated the efficacy of pembrolizumab compared to standard treatment (chemotherapy ± bevacizumab or cetuximab) as a first-line treatment for MSI-H/dMMR-type mCRC patients, with results showing a median PFS extension from 8.2 months to 16.5 months. The CheckMate142 study (27) included a total of 45 previously untreated mCRC patients who received 3 mg/kg nivolumab q2w + 1 mg/kg low-dose ipilimumab q6w, with an ORR of 69%, a CR of 13%, a 2-year PFS rate of 74%, and a 2-year OS rate of 79%. Immune monotherapy or dual immune combination therapy has become a new first-line standard treatment for MSI-H mCRC, but immunotherapy for MSS colon cancer is almost ineffective, as these patients are considered to have cold tumors with minimal lymphocyte infiltration (8). Transforming cold tumors into hot tumors can greatly enhance the efficacy of immunotherapy. The growth and invasion of tumors depend on the generation of blood vessels (28). In 1971, Folkman (29) proposed an important connection between solid tumors and capillaries: after solid tumors are formed, they induce the proliferation of endothelial cells in the surrounding blood vessels, leading to the formation of new capillaries. Moreover, in the absence of new capillaries, the vast majority of solid tumors will further cease to grow, illustrating the importance of new capillaries in the growth of solid tumors. Subsequently, Brem et al. (30) confirmed that the invasion of solid tumors depends on the presence of neovascularization. Bevacizumab, as a recombinant human monoclonal antibody against vascular endothelial growth factor (VEGF), has been proven to specifically bind to VEGF and block its binding to receptors, not only affecting the generation of new blood vessels but also leading to the degeneration of existing blood vessels, thereby further inhibiting tumor growth. This antagonistic effect can also normalize blood vessels, thereby increasing the rate of drug delivery within the tumor, achieving the goal of further controlling tumor growth. The combined application of chemotherapy can improve the efficacy of chemotherapy. The NICHE (31) study is the first clinical study to explore dual immunotherapy neoadjuvant treatment in patients with stage I-III colorectal cancer. The study results showed that a portion of pMMR patients who were enrolled also showed treatment responses, with 27% of patients achieving pathological response, 20% achieving major pathological response (MPR), and 13.3% achieving pathological complete response (pCR), indicating that MSS patients may also benefit from immunotherapy (32). In the REGONIVO study (5), the combination of regorafenib and nivolumab was used to treat patients with advanced metastatic colorectal cancer, showing encouraging anti-tumor activity, with an ORR of 33% and a median PFS of 7.9 months in MSS mCRC patients. The REGOTORI study (6, 33), a phase Ib/II clinical study, aimed to evaluate the safety, tolerability, and preliminary efficacy of regorafenib combined with toripalimab in MSS mCRC patients who had failed or could not tolerate systemic chemotherapy. This study demonstrated good efficacy, with an ORR of 15.2% and a DCR of 36.4%, providing evidence supporting the improvement of immunogenicity in the tumor microenvironment through the combination of anti-angiogenic drugs and immunotherapy. In this study, we found that there was a statistically significant difference in the percentage of CD8+ T lymphocytes, TAMs, and CAFs between the two groups after treatment. The experimental group had more patients with increased numbers of CD8+ T lymphocytes and decreased numbers of TAMs and CAFs after treatment than did the control group. To further validate our results, we introduced the immunohistochemistry score. Comparing the post-treatment immunohistochemistry scores of CD8+ T lymphocytes, TAMs, and CAFs between the two groups showed significant differences (*P<0.05*). This was primarily reflected in the increased number of patients with CD8+ T lymphocytes immunohistochemistry scores ≥ 2 and the decreased number of patients with TAMs and CAFs immunohistochemistry scores ≥ 3 and ≥ 4, respectively. These changes were more pronounced in the experimental group. Additionally, comparing the pre- and post-treatment immunohistochemistry scores of CD8+ T lymphocytes, TAMs, and CAFs between the two groups revealed statistically significant differences in the experimental group (*P<0.05*). This indicates that patients in the experimental group experienced a significant increase in the infiltration of immune cells within the tumor after combined treatment, enhancing the body’s antitumor capability, inhibiting tumor invasion and metastasis, and improving treatment efficacy. The reasons for this may include the following: Firstly, sintilimab can reactivate and activate the human immune system. With a strong affinity for the PD-1 receptor, it exerts immunoregulatory and antitumor effects at the immune checkpoint PD-1. Its slow dissociation rate allows it to produce a lasting and stable antitumor effect, effectively blocking tumor cell signal transduction and promoting tumor cell apoptosis. Sintilimab also activates suppressed antitumor immune cells, restores T lymphocyte function, enhances the cytotoxic effect of T lymphocytes on tumor cells, and reduces DNA mismatch repair, thereby maintaining genomic stability (34). Secondly, by directly acting on human T lymphocytes, sintilimab exhibits a strong antitumor effect, enabling normal secretion of immune cells such as CD4+, CD8+, and CD4+/CD8+. It also enhances the immune response of T cells against cancer cells, indirectly contributing to the antitumor effect; Finally, studies (35, 36) have shown that there is an important connection between the tumor immune microenvironment and tumor angiogenesis. Inhibiting the formation of blood vessels can improve the tumor immune microenvironment, promote the infiltration of more T lymphocytes into the tumor, transform the immunosuppressive state of cold tumors into an immune-supportive state of hot tumors, and greatly enhance the effectiveness of immunotherapy. Immune checkpoint inhibitors can not only normalize tumor blood vessels (37). By specifically recognizing the binding sites of PD-1 and PD-L1 and blocking them, the ability of immune cells to distinguish tumor cells is restored, thereby enhancing the body’s antitumor ability (38). Therefore, the combined use of ICIs and antiangiogenic drugs may achieve a synergistic effect in tumor treatment. This may also be the internal reason why the experimental group had greater PFS, mPFS, ORR, and DCR than did the control group.

According to CTCAE, any grade adverse events that occurred during the treatment of all patients in both groups were recorded. The adverse events in both groups were of Grade I or II, with no Grade III or higher adverse events reported. Furthermore, there were no cases of death due to drug-related adverse reactions in either group. All Grade I and II adverse events were effectively controlled through active management of drug adverse reactions, and no patients experienced drug discontinuation or dose reduction. The specific adverse reactions in the two groups were as follows: In the experimental group, 3 cases of hypertension were effectively controlled with oral antihypertensive medication, while no cases of hypertension were observed in the control group. A total of 9 patients in both groups developed proteinuria, which was effectively managed through active renal protection and symptomatic treatment. The experimental group had 1 case of epistaxis, which was effectively controlled by short-term compression and did not recur during subsequent treatments. Both groups experienced bone marrow suppression, mainly mild to moderate leukopenia and thrombocytopenia, which were effectively controlled with medications to increase white blood cells and platelets. Hypothyroidism occurred in patients from both groups, but it improved with close monitoring of thyroid function and supplementation with levothyroxine, without affecting the continuation of medication. Immune enteritis was observed in both groups after medication, but it was effectively controlled with fluid supplementation, oral antidiarrheal drugs, and symptomatic supportive treatment to maintain electrolyte balance. Rash adverse reactions were managed effectively with anti-allergic symptomatic treatment in both groups. There was no significant difference in the incidence of adverse events and the total incidence of adverse events between the two groups (*P>0.05*). This indicates that, compared to the control group, the experimental group not only improved the therapeutic effect of the medication but also ensured the safety of the patients, with drug-related adverse reactions being controllable. Therefore, for patients with advanced colorectal cancer undergoing second-line treatment, especially those with decreased chemotherapy tolerance, elderly and frail patients, and those with significant cumulative toxicity from chemotherapy and disease complications, this treatment regimen may be safer and more effective.

In the short-term follow-up after treatment of the two groups of patients, we found that there were deaths in both groups. There were a total of 8 deaths in the experimental group, for a survival rate of 86.70%. Among them, 5 deaths were due to cachexia, and 3 deaths were due to liver metastasis leading to liver failure. In the control group, there were a total of 11 deaths, for a survival rate of 78.00%. Among them, 6 deaths were due to cachexia, 3 deaths were due to liver metastasis leading to liver failure, 1 death was due to delayed treatment of secondary complete intestinal obstruction resulting in severe infection, and 1 death was due to brain metastasis causing brain herniation. Survival curves showed a rightward shift in the experimental group compared to the control group, indicating that the PFS of deceased patients in the experimental group was significantly longer than that in the control group (*P<0.05*). This further suggests that the application of ICIs combined with antiangiogenic drugs can inhibit tumor growth, infiltration, and metastasis, leading to improved PFS in patients. When analyzing the prognostic factors affecting second-line treatment of MSS/pMMR advanced colorectal cancer patients in this study using Cox regression models, we found that patients over 60 years old, female patients, and patients in the control group had a higher risk of death compared to patients aged ≤60 years, male patients, and patients in the experimental group. The risk of death for patients aged ≤60 years, male patients, and those in the experimental group was only 15.30%, 30.50%, and 25.70%, respectively, compared to patients aged >60 years, female patients, and those in the control group. This indicates better treatment response for these patient groups in this study. Although Cox regression analysis in this study showed that tumor differentiation and clinical stage did not significantly affect the prognosis of second-line treatment in MSS/pMMR advanced colorectal cancer patients, we found through our study data that patients with poorly differentiated and moderately differentiated tumors and clinical stage IV had a higher risk of death compared to patients with well-differentiated tumors and clinical stage III, suggesting poorer treatment response for these patient groups in this study. Both groups included patients with liver metastases from colorectal cancer, and this difference was not statistically significant (*P>0.05*). However, liver metastasis can undeniably reduce the efficacy of immunotherapy (39). How to overcome liver immune tolerance mechanisms and their adverse effects so that patients with colorectal cancer and liver metastases can benefit more from immunotherapy is worth further exploration in the future.

## Conclusions

The combination therapy of ICIs and antiangiogenic drugs can not only improve the tumor immune microenvironment of patients with MSS/pMMR advanced CRC who have failed first-line treatment but also promote the transformation of a cold tumor immune suppression state into a hot tumor immune supportive state. On the premise of ensuring the safety of adverse reactions to drugs, it enhances the antitumor efficacy of patients and clinical treatment effects, further increasing its clinical application value.

This study has certain limitations: firstly, it is a prospective single-center, and second-line treatment study, and although strict inclusion, exclusion, and withdrawal criteria were formulated, there may still be some degree of selection bias in the sample population; secondly, the sample size of patients in the experimental group in this study is small, only 60 cases; finally, the follow-up time for patients is limited, and survival time is not yet mature, limiting long-term efficacy and prognosis. Therefore, larger sample size and multicenter prospective studies are still needed to provide more evidence-based medicine evidence for the efficacy of pembrolizumab combined with bevacizumab in treating MSS/pMMR advanced CRC patients who have failed first-line treatment, thereby promoting clinical diagnosis and treatment of advanced CRC.

## Data Availability

The raw data supporting the conclusions of this article will be made available by the authors, without undue reservation.
